# Correlation between Metabolite of Prostaglandin E2 and the incidence of colorectal adenomas

**DOI:** 10.3389/fonc.2023.1068469

**Published:** 2023-02-27

**Authors:** Jia Jiang, Anjie Li, Xiaolian Lai, Hanqun Zhang, Chonghong Wang, Huimin Wang, Libo Li, Yuncong Liu, Lu Xie, Can Yang, Cui Zhang, Shuoyan Lu, Yong Li

**Affiliations:** ^1^ Guizhou University of Traditional Chinese Medicine, Guiyang, China; ^2^ Department of Medicine-Cardiovascular, Guizhou Provincial People’s Hospital, Guiyang, China; ^3^ Department of Digestive, People's Hospital of Songtao Miao Autonomous County, Tongren, China; ^4^ Department of Oncology, Guizhou Provincial People’s Hospital, Guiyang, China; ^5^ Zunyi Medical University, Zunyi, China

**Keywords:** colorectal adenoma, prostaglandin E2, PGE-M, Colorectal cancer, cancer risk, bio-markers

## Abstract

Colorectal cancer is a common malignancy, and the incidence and mortality rates continue to rise. An important factor in the emergence of inflammation-induced colorectal carcinogenesis is elevated cyclooxygenase-2. Prostaglandin E2 (PGE_2_) over-production is frequently equated with cyclooxygenase-2 gene over-expression. PGE_2_ can be assessed by measuring the level of prostaglandin’s main metabolite, PGE-M, in urine. Colorectal adenoma is a precancerous lesion that can lead to colorectal cancer. We conducted research to evaluate the association between urinary levels of the PGE-M and the risk of colorectal adenomas. In a western Chinese population, we identified 152 cases of adenoma and 152 controls patients without polyps. Adenoma cases were categorized into control, low-risk and high-risk groups. There was no significant change in PGE-M levels, between the control group and the low-risk adenoma group. In the high-risk group, the PGE-M levels were 23% higher than the control group. When compared to people with the lowest urine PGE-M levels (first quartile), people with greater urinary PGE-M levels had a higher chance of developing high-risk colorectal adenomas, with an adjusted odds ratio (95% CI) of 1.65 (0.76-3.57) in the fourth quartile group, (p= 0.013). We conclude urinary PGE-M is associated with the risk of developing high-risk adenomas. Urinary PGE-M level may be used as a non-invasive indicator for estimating cancer risk.

## 1 Introduction

Colorectal cancer incidence around the world has increased in tandem with an increase in respective Human Development Indices. This disease has the third highest incidence rate and the second highest fatality rate, globally (1).Similarly, colorectal cancer is becoming more common in China (2). Numerous investigations on the adenoma-carcinoma sequence have conclusively shown that between 60% and 90% of sporadic colorectal cancers result from adenomas that have undergone malignant transformation (3, 4). Therefore, the risk of cancer can be decreased by identifying risk factors for adenomas and preventing their growth (5).

Increasing evidence points to the possibility that inflammation increases the vulnerability of developing colorectal malignancies (6, 7). The enzyme cyclooxygenase-2 (COX-2), mediates the relationship between cancer and inflammation and is 50-85% more abundant in patients with colorectal malignancies (8, 9). COX-2 is a key rate-limiting enzyme for the conversion of arachidonic acid to prostaglandins (PG), and when COX-2 gene expression is elevated, more prostaglandin E2(PGE_2_) is produced (10, 11). Clinical studies have demonstrated that non-steroidal anti-inflammatory drugs (NSAIDs) lower the chance of developing adenoma and colorectal tumors, and their effects are linked to the suppression of prostaglandin 2 and cyclooxygenase-2 (12–15). The pro-inflammatory mediator PGE_2_ has been shown to be able to support colorectal tumor progression through a variety of mechanisms. PGE_2_’s primary effects include inhibiting apoptosis, promoting angiogenesis, and encouraging epithelial cell proliferation、 survival、 migration、invasion、repair and regeneration (16–20).It is the most abundant prostaglandin found in colorectal cancer patients (21).

Multiple lines of research indicate that COX-2-derived PGE_2_ has a role in the growth of colorectal adenomas and can predict the risk of developing colorectal cancer (22). However, epidemiological evidence directly linking urinary PGE_2_ levels to the risk of colorectal adenoma is lacking in China. Direct measurement of unstable PGE_2_, however, is unreliable. Currently the best method for measuring systemic PGE_2_ synthesis *in vivo* to assess the quick metabolism of PGE_2_ by 15-hydroxyprostaglandin oxidase to form stable 11 alpha-hydroxy-9,15dioxo-2,3,4,5-tetranorprostane-1,20-dioicacid (PGE-M) (23). A nested study design has shown a connection between Chinese women’s urinary PGE-M levels and their chance of developing colorectal carcinomatosis (24). By examining samples and data gathered from colorectal adenoma patients and a healthy control population in Guizhou Provence, the present study evaluates the relationship between urinary PGE-M levels and the risk of colorectal adenoma in a in western China population. This study also aims to provide new strategies and tools for implementing interventions in the early stages of tumors development.

## 2 Materials and methods

### 2.1 Study population

Study participants were chosen from the Guizhou Cancer Center, Guizhou Provincial People’s Hospital, and Songtao Miao Autonomous County People’s Hospital in Guizhou province to screen for colorectal adenomas. Patients were initially seen at the endoscopy centers of the aforementioned hospitals. Participants were 18-75 years of age and in generally good health with no vital organ failures. According to WHO guidelines, a colorectal adenoma diagnosis was made, and the degree of neoplasia was assessed. The following criteria was used to diagnosis colorectal adenoma: ①Adenoma was verified by a pathological biopsy, ② villous adenoma or mixed adenoma with more than 25% villous-like features was identified, ③high-grade epithelial neoplasia was identified. Patients with colorectal adenoma who met the aforementioned diagnostic standards had a subsequent colonoscopy adenectomy, and histology was used to confirm the diagnosis. All specimens were examined by two or more experienced pathologists in the hospital’s pathology department. Exclusion criteria for our study included those with a history of familial adenomatous polyposis (FAP), inflammatory bowel disease, hereditary non-polyposis colorectal cancer (HNPCC), Turcot syndrome, severe cardiovascular disease, recurrent adenoma with a confirmed diagnosis, colorectal cancer, and tumors in other organs. Because NSAIDs affect PGE-M levels, subjects had used aspirin or any NSAID for at least 48 hours prior to colonoscopy. They were ineligible for analysis. Our study also excluded participants. Because they used any dose of NSAIDs, including aspirin, for 3 days or more in the 3 months prior to enrollment or 3 days per month or used NSAIDs for 36 days in the past year. Finally, a sample of 304 participants’ data was kept for analysis. The study protocol was approved by the Ethics Committee of Guizhou Provincial People’s Hospital. A signed informed consent was required for all study subjects.

### 2.2 Sample collection

There were 412 study participants who provided urine samples; however only 304 samples were usable, due to sample damage and inconvenience of follow-up during the novel coronavirus pandemic. There were 152 cases identified as negative controls. This meant they did not have any polyps at the endoscopic screening. Patients with single tubular adenomas with a maximum diameter of less than 1 cm were categorized as low-risk cases, whereas those with a maximum diameter of more than 1 cm and/or histology of tubular villi, villi, and any multiple adenomas were categorized as high-risk cases (22, 25, 26). **Table 1** displays the characteristics of the three categories. To stop the oxidation of unstable metabolites, urine samples were taken in sterile cups containing 125 mg of ascorbic acid. Following collection, samples were kept chilled (at about 0 to 4°C) in a portable foam box with an ice pack before being processed within 6 hours for long-term storage at -80 ± 5°C. Each participant had a biospecimen collection form filled out at the time of sample collection, which listed the day and time of sample collection as well as any drug usage within the previous 48 hours of the colonoscopy.

**Table 1:** Baseline characteristics of colorectal adenoma cases and matched controls.

Baseline Characteristics	Control	Case		p^a^
		Low-risk adenoma	High-risk adenoma	
n	152	59	93	
Age, mean(SD),years	57.48(12.69)	60.17(10.09)	61.95(8.81)	0.009
Sex, male (%)	65.8	69.5	75.3	0.307
Education, <high school (%)	58.6	64.4	54.8	0.518
Ever smoked regularly (%)	53.3	64.4	61.3	0.249
Ever drank regularly (%)	20.4	25.4	26.9	0.469
History of hypertension (%)	30.3	28.8	45.2	0.035
History of diabetes (%)	9.9	10.2	20.4	0.043
BMI, mean(SD), kg/m^2^	25.77(6.21)	23.96(3.34)	25.89(6.69)	0.104
Calcium intake, mean(SD),g/d	664.73(243.32)	657.20(185.95)	707.71(266.01)	0.317

P^a^ values were obtained by chi-square test for categorical variables, and analysis of variance for age, BMI, and calcium intake.

We developed the questionnaire for this study based on the food frequency questionnaire (SQFFQ) used in the 2002 Chinese population dietary survey methodology, and made appropriate adjustments to incorporate the regional dietary habits of Guizhou. All participants completed the questionnaire at the time of study enrollment. The first section evaluates the annual average food consumption, including average intake, frequency of consumption, etc.; the second section tracks nutrient supplement usage, including the name of the supplements, dose, and regimen. The Chinese Food Composition Table (2nd edition) and Nutrition Calculator V2.7.3 were used to convert nutrient intakes, to evaluate the overall amount of calcium consumed through diet and dietary supplements. Information on medical history, drug usage, demographics, anthropometrics, daily food habits, physical activity, and other lifestyle factors were also included as added content to this questionnaire.

### 2.3 Laboratory measurement

PGE-M was measured using liquid chromatography/tandem mass spectrometry to determine the endogenous production of PGE_2_ in humans (27). Briefly, urine was placed in a 10 mL polypropylene tube at room temperature, and then a sample of 1.0 mL was acidified to pH=3 by adding 1.0 mol/L HCl. Next, endogenous PGE-M was converted to methoxime derivatives and treated with 1600 mg of methoxamine hydrochloride in 10 mL of 1.5 mol/L sodium acetate solution (pH=5). The methoxylated PGE-M was dissolved in 8 ml of water after 1 hour of greenhouse incubation, and the aqueous sample was then transferred to C-18 Sep-Pak that had been prepared with 5 ml of methanol and 5 ml of water (pH 3). Sep-Pak was then eluted with ethyl acetate. Thermo SCIENTIFIC Hypersil GOLD (1.9 µm, 2.1×50 mm) column linked to a TSQ-Altis, and Thermo Fisher mass spectrometry pump was then used for liquid chromatography. Heated electrospray ion source was used as the ionization technique. The mass to charge ratios (m/z) monitored were 385.3 ~ 336 and m/z 385.3 ~ 367 for endogenous PGE-M, in the selected response monitoring (SRM) mode. The ratio of the mass spectral peak regions of the m/z 336 and m/z 367 ions was used to calculate the amount of endogenous PGE-M. With a coefficient of variation of 4.1% between batches and 8.7% within batches, the lower limit of detection for PGE-M was set at 2.00 ng/ml. There were no incidents that compromised data integrity or quality throughout the experiment. The quality control samples’ identities and the status of the urine samples used in the study were both unknown to the laboratory staff. Additionally, Urinary creatinine was measured using a Sigma kit (Sigma Co., Inc., St. Louis, MO, USA). The levels of urinary creatinine were determined and reported as standardized PGE-M values, PGE-M (ng)/creatinine (mg).

### 2.4 Statistical methods

Selected baseline characteristics for cases and controls were computed as means, standard deviations, and percentages. We compared the means of age, body mass index (BMI), and calcium intake data between case and control participants using analysis of variance. To compare categorical variables, we employed the chi-square test. Urinary PGE-M levels for each sample were normalized using the urinary creatinine level of the sample and expressed as ng/ml creatinine. The PGE-M data in urine were skewed to the right; therefore, the median, interquartile range, and geometric mean were estimated for descriptive statistics. After adjusting for age, sex, smoking status, alcohol use, education, and prior hypertensive diabetes mellitus, Wilcoxon rank-sum tests and log-transformed linear regression models were used to analyze differences in PGE-M levels between groups. The PGE-M concentrations in the control group’s quartile distribution served as the basis for establishing cut points for categorical variables. The odds ratio (OR) and 95% confidence interval (95% CI) between urine PGE-M levels and the risk of colorectal adenoma were calculated using logistic regression models. Trend p-values were derived by using categorical variables as continuous parameters of the model and passing the linear trend test. We also stratified associations by subgroups, such as BMI, sex and calcium intake level, in order to focus on these factors’ influence on the association of urinary PGE-M levels with the incidence of high-risk colorectal adenomas. We performed all analyses using SPSS 26.0 (SPSS Inc, Chicago, IL, USA), and P values ≤0.05 (two-sided probability) were interpreted as statistically significant for all analyses.

## 3 Results

### 3.1 Baseline characteristics

In this investigation, 304 patient urine samples were examined. We found that the sample group had 153 controls, 59 low-risk, and 93 high-risk cases. **Table 1** displays the characteristics of the cases and the controls. The high-risk adenoma group had a greater prevalence of diabetes mellitus, hypertension, and a higher BMI when compared to the control group. Low-risk adenomas were more prevalent in people with lower education levels and low calcium intake. In contrast to controls, patients with adenomas were more likely to be male, smokers, and drinkers, although the difference was not statistically significant.

### 3.2 Baseline levels of urinary PGE-M

**Table 2** shows the baseline urinary PGE-M levels. Urinary PGE-M levels in the low-risk adenoma patients’ group did not differ statistically from those in the control group. However, urinary PGE-M levels were higher in individuals with high-risk adenomas. patients with high-risk adenomas had urinary PGE-M levels 7% and 23.56% higher than patients with low-risk adenomas or control group patients, respectively (p=0.04).

**Table 2:** Baseline levels of urinary PGE-M (ng/mg creatinine) in cases and controls.

Study Group	n	Median (Q1, Q3)	Difference (%)	p^a^	Geometric mean(95%CI)	Difference (%)	p^b^
Controls	152	10.54(5.73,16.66)			9.76(11.25-14.76)		
Cases							
High-risk adenoma	93	11.28(6.07,25.41)	7.02	0.11	12.06(13.71-21.84)	23.56	0.04
Low-risk adenoma	59	8.70(6.11,15.79)	17.46	0.51	9.08(9.27-12.97)	6.97	0.68

P^a^ values were calculated by Wilcoxon signed rank test.

P^b^ Linear regression models from log-transformed PGE-M levels, adjusted for age, sex, smoking status, alcohol consumption, education, and previous hypertensive diabetes.

Difference= (geometric mean or median of risk groups - geometric mean or median of control groups)/(geometric mean or median of control groups).

### 3.3 Association between urinary PGE-M and incidence of colorectal adenoma

The Spearman correlation coefficients between urine PGE-M level and several lifestyle factors are shown in **Table 3**. The results showed a direct correlation between PGE-M and age, gender, and smoking status. We further analyzed urinary PGE-M levels and the risk of developing colorectal adenomas (**Table 4**). High-risk adenomas were more likely to occur in patients with higher urine PGE-M concentrations (p =0.013-0.016). The highest, fourth quartile, urine PGE-M levels were associated with a 1.65-fold higher incidence of high-risk colorectal adenoma compared to the lowest urinary PGE-M levels. Results did not indicate an association between urine PGE-M levels and an increased incidence of low-risk adenomas.

**Table 3:** Spearman’s correlation coefficient between urinary PGE-M and several lifestyle factors.

Variable	PGE-M	p
PGE-M (ng/mg Cr.)	1.00	
Age	-0.126	0.029
Sex, male	0.215	<0.001
BMI (kg/m^2^)	0.085	0.142
Ever smoked regularly	0.115	0.045
Ever drank regularly	0.046	0.423
History of hypertension	0.027	0.642
History of diabetes	0.068	0.234
Calcium intake(mg/d)	0.066	0.250

**Table 4:** The relationship between baseline urinary PGE-M levels and incidence of colorectal adenoma.

Study Group	PGE-M(quartile)				p for trend
	Q1(low)	Q2	Q3	Q4	
Controls					
n	38	38	38	38	
High-risk					
n	19	27	14	33	
OR (95%CI) ^a^	1.00(reference)	1.42(0.69-2.98)	0.74(0.32-1.68)	1.74(0.85-3.58)	0.016
OR (95%CI) ^b^	1.00(reference)	1.16(0.53-2.52)	0.75(0.31-1.80)	1.65(0.76-3.57)	0.013
Low-risk					
n	12	22	12	13	
OR (95%CI) ^a^	1.00(reference)	1.83(0.80-4.23)	1.00(0.40-2.50)	1.08(0.44-2.68)	0.222
OR (95%CI) ^b^	1.00(reference)	1.80(0.76-4.28)	1.00(0.38-2.63)	1.09(0.42-2.63)	0.321

a: OR and 95 CIs from conditional logistic models.

b: OR and 95 CIs from conditional logistic models corrected for BMI, smoking, alcohol consumption, education, hypertension, and diabetes

### 3.4 Association of urinary PGE-M level with incidence of high-risk colorectal adenoma, stratified by BMI, gender, and calcium intake

Urinary PGE-M levels were tested in relation to the incidence of high-risk colorectal adenoma, stratified by BMI, gender, and calcium intake (**Table 5**). Within the highest PGE level, fourth quartile, PGE level subgroup, women had a stronger association with the incidence of high-risk colorectal adenomas (adjusted OR, 3.72, 95% confidence interval, 0.54-25.45) than did males (adjusted OR, 1.75; 95% confidence range, 0.66-4.61). Although not statistically significant, the patient subgroup with BMI ≥25 (kg/m^2^) had OR changing from 1.0 to 3.46, to 1.50, to 2.30 for each increasing PGE-M quartiles respectively. Body weight may modify the association between urinary PGE-M and incidence of high-risk colorectal adenoma. The present study did not find a significant interaction between BMI, gender, calcium intake and PGE-M in the high-risk adenoma group (P for interaction >0.05).

**Table 5:** Association of urinary PGE-M levels with incidence of high-risk colorectal adenoma stratified by BMI, gender, and calcium intake.

	PGE-M(quartile)				p for trend	P for interaction
	Q1(low)	Q2	Q3	Q4		
BMI<25( kg/m^2^)						
Case/Controls	14/18	12/24	7/18	21/22	0.82	0.11
OR (95%CI) ^a^	1.00(reference)	0.49(0.17-1.39)	0.44(0.13-1.41)	1.25(0.48-3.26)		

BMI≥25( kg/m^2^)						
Case/Controls	5/20	15/14	7/20	12/16	0.57	
OR (95%CI) ^a^	1.00(reference)	3.46(0.91-13.16)	1.50(0.33-6.76)	2.30(0.58-9.20)		
Males						
Case/Controls	11/19	23/21	9/30	27/30	0.12	0.05
OR (95%CI) ^a^	1.00(reference)	1.90(0.70-5.16)	0.52(0.17-1.63)	1.75(0.66-4.61)		

Females						
Case/Controls	8/19	4/17	5/8	6/8	0.12	
OR (95%CI) ^a^	1.00(reference)	0.19(0.02-1.38)	0.56(0.06-5.25)	3.72(0.54-25.45)		
Calcium intake<613(mg/d)^b^						
Case/Controls	8/18	15/18	2/18	15/22	0.09	0.12
OR (95%CI) ^a^	1.00(reference)	2.10(0.63-7.02)	0.21(0.03-1.43)	1.38(0.42-4.60)		
Calcium intake>613(mg/d)^b^						
Case/Controls	11/20	12/20	12/20	18/16	0.77	
OR (95%CI) ^a^	1.00(reference)	0.92(0.30-2.80)	1.32(0.43-4.01)	2.01(0.68-5.94)		

a: OR and 95% CI from conditional logistic models corrected for BMI, smoking status, alcohol consumption, education, hypertension, and diabetes.

b: Median calcium intake of the control group was taken

## 4 Discussion

In the current investigation, we discovered a positive correlation between high urine PGE-M levels and the incidence of high-risk colorectal adenomas, but no such correlation was found for single tubular adenomas smaller than 1 cm. These findings imply that urine PGE-M level may be a helpful non-invasive indicator of adenoma risk. Additionally, we discovered that individuals with above-average or low body weight had a strong correlation with PGE-M.

Numerous pieces of evidence point to colorectal adenoma as a prominent precancerous condition of the colorectum. Colorectal adenoma has a complicated etiology, involving a number of biological pathways, one of which has been demonstrated to be COX-2 (8, 9). Arachidonic acid is one of the fatty acid substrates that are converted by COX-2, an inducible isoform of COX, into pro-inflammatory prostaglandins (7). PGE_2_ is a crucial mediator of the proto-oncogenic actions of COX (28).Therefore, COX-2 inhibitor use lowers urine PGE_2_ levels, which lowers the incidence of colorectal cancers and adenomas (12, 13). The primary urinary PGE_2_ metabolite is PGE-M. Additionally, earlier research by others has demonstrated an association between the down-regulation of 15-Hydroxyprostaglandin dehydrogenase (15-PGDH) expression and activity in colorectal cancer and the generation of the urinary PGE_2_ metabolite PGE-M (29–31).

It was found in a prospective trial of Chinese women that baseline urine PGE-M was most likely a urinary marker for early prediction of CRC and was linked to a high likelihood of advanced colorectal cancer diagnosis (24). In several earlier investigations, it was discovered that patients with advanced adenomas and numerous tubular adenomas had much greater PGE-M levels than the controls group patients (26, 32, 33). Therefore, based on the quantity, size, and complexity of the adenomas, we categorized the cases in this study into high-risk, low-risk and control groups. Our results confirmed this association between PGE-M and high-risk adenomas. We observed higher levels of urinary PGE-M in the western Chinese population in the high-risk of adenoma group, compared to the low-risk group. Calcium may control the inflammatory response affecting colorectal adenomas by affecting a variety of mechanisms including bile acid catabolism, immune regulation, and fatty acid metabolism (34). The present study also considered the influence of calcium intake. However, only the potential effect of calcium intake on correlation among US registered female nurses was assessed in the relevant study (p>0.05) (22). Our results are in line with those of earlier research, although the associations between PGE-M level and the low and high-risk groups was not significantly changed by calcium intake levels. This is the first epidemiological study of calcium intake levels, urinary PGE-M levels, and risk of colorectal adenoma in western China. Currently, calcium is still one of the most deficient nutrients among Chinese residents, and this situation may be even more serious in western China (35). We hypothesize that high levels of PGE_2_ may be determined by a combination of calcium deficiency and individual genetic susceptibility. Therefore, further studies are needed to explore and reveal the mechanisms and significance of calcium intake levels related to PGE_2_ and colorectal adenoma, and identify key genes for calcium re-absorption.

In this study, we had several advantages. These included the random recruitment of participants in a large sample database at the Guizhou Cancer Center. This allowed us to collect patient urine samples before diagnosis and avoid selectivity bias. All participants received an endoscopy, and all adenomas excised from the case group underwent a pathological evaluation, which contributed to the accuracy of the groups. The Tennessee Colorectal Polyp Study in the United States has conducted a number of studies on PGE-M and the risk of acquiring colorectal adenomas (26, 32). To further these earlier findings, the present study was carried out in a population in western China. For the first time in China, we used liquid chromatography/tandem mass spectrometry to detect PGE-M levels. It must be acknowledged that our study also has many limitations. For example, our sample size was constrained after relevant cases were excluded due to individual differences or other factors like the use of NSAIDs. Second, the long-term association between urinary PGE-M levels and the risk of developing colorectal adenoma needs to be further studied because, further studied only measured the PGE-M levels from urine samples at one time point. Although we studied calcium intake level and PGE-M levels’ influence on the incidence of colorectal adenoma, we could not assess how calcium deficiency alters urinary PGE-M level and colorectal adenoma incidence. A more comprehensive understanding of colorectal adenoma development at the molecular and genetic level is needed.

In conclusion, this study evaluated the association of urinary PGE-M levels with increased incidence of high-risk colorectal adenomas in a western Chinese population. Because high-risk adenomas have the greatest likelihood of malignant development, it may be possible to intervene early and prevent cancer by using urine PGE-M levels as a non-invasive indicator for estimating cancer risk.

## Data availability statement

The original contributions presented in the study are included in the article, further inquiries can be directed to the corresponding authors.

## Ethics statement

The studies involving human participants were reviewed and approved by the Ethics Committee of Guizhou Provincial People’s Hospital. The patients/participants provided their written informed consent to participate in this study.

## Author contributions

(I) Conception and design: YoL, SL. (II) Administrative support: YoL, AL, HZ. (III) Provision of study materials or patients: HW, CW, LL. (IV) Collection and assembly of data: XL, JJ, YuL. (V) Data analysis and interpretation: XL, JJ, XL, CY, CZ. (VI) Manuscript writing: All authors. (VII) Final approval of manuscript: All authors. All authors contributed to the article and approved the submitted version.
